# Prolonged febrile seizures induce inheritable memory deficits in rats through DNA methylation

**DOI:** 10.1111/cns.13088

**Published:** 2019-01-21

**Authors:** Yun‐Jian Dai, Deng‐Chang Wu, Bo Feng, Bin Chen, Yang‐Shun Tang, Miao‐Miao Jin, Hua‐Wei Zhao, Hai‐Bin Dai, Yi Wang, Zhong Chen

**Affiliations:** ^1^ Institute of Pharmacology & Toxicology, NHC and CAMS Key Laboratory of Medical Neurobiology, College of Pharmaceutical Sciences Zhejiang University Hangzhou China; ^2^ Department of Pharmacy, Second Affiliated hospital, School of Medicine Zhejiang University Hangzhou China; ^3^ Department of Pharmacy School of Medicine Children’s hospital Zhejiang University Hangzhou China; ^4^ Epilepsy Center, Department of Neurology School of Medicine Second Affiliated Hospital Zhejiang University Hangzhou China

**Keywords:** DNA methylation, febrile seizures, memory, transgenerational transmission

## Abstract

**Aims:**

Febrile seizures (FSs) are the most common types of seizures in young children. However, little is known whether the memory deficits induced by early‐life FSs could transmit across generations or not.

**Methods:**

The memory functions of different generations of FS rats were behaviorally evaluated by morris water maze, inhibitory avoidance task, and contextual fear conditioning task. Meanwhile, molecular biology and pharmacological methods were used to investigate the role of DNA methylation in transgenerational transmission of memory defects.

**Results:**

Prolonged FSs in infant rats resulted in memory deficits in adult and transgenerationally transmitted to next generation, which was mainly through mothers. For these two generations, DNA methyltransferase (DNMT) 1 was upregulated, leading to transcriptional inhibition of the synaptic plasticity protein reelin but not the memory suppressor protein phosphatase 1. DNMT inhibitors prevented the high expression of DNMT1 and hypermethylation of *reelin* gene and reversed the transgenerationally memory deficits. In addition, enriched environment in juvenile rats rescued memory deficits induced by prolonged FSs.

**Conclusions:**

Our study demonstrated early experience of prolonged FSs led to memory deficits in adult rats and their unaffected offspring, which involved epigenetic mechanisms, suggesting early environmental experiences had a significant impact on the transgenerational transmission of neurological diseases.

## 1 INTRODUCTION

Behavioral and emotional disorders acquired from early‐life adverse environmental experiences can be transmitted to future offspring.^1^, ^2^ These transgenerational transmissions are intriguing and important. Memory, one of the most important abilities, can be impaired by neurological disorders induced by environmental stimulation in early life.^3^ In addition, juvenile‐enriched environment can improve memory formation in both the normal and diseased state, which has been observed in the affected generation as well as in their unaffected offspring.^4^ However, little is known whether the memory deficits affected by early‐life neurological disorders could transmit across generations or not.

Febrile seizures (FSs) induced by early‐life hyperthermia are the most common diseases in childhood.^5^, ^6^ As previously reported that (a) recognition memory impairment is detected after FSs in clinic^7^; (b) deficits in hippocampal working memory^8^ and long‐term memory of inhibitory avoidance task^3^ have also been demonstrated in animal studies. Notably, most studying the cognitive consequences of FS have typically focused on one generation; however, we have previously reported that enhanced susceptibility to seizures after acquired FS can be transgenerationally transmitted to their unaffected offspring.^9^ We are eager to know whether these cognitive outcomes after FSs can be transmitted across generations or not.

Recently, growing evidence has shown that environmental exposure influences transgenerational epigenetic changes^10^, ^11^ and subsequently results in phenotypes across generations.^12^ Therefore, we investigated whether DNA methylation, a main composition of epigenetic modification, participated in the memory deficits induced by serious FSs in affected adult rats and their offspring. Additionally, we examined whether enriched environment other than pharmacotherapy could rescue memory deficits induced by infant FSs experience.

## 2 MATERIALS AND METHODS

### 2.1 Experimental animals

Sprague‐Dawley rats were used in this study (Experimental Animal Center, Zhejiang University, China). They were maintained in cages with a 12 hours light‐dark cycle (lights on from 8:00 to 20:00) with free access to food and water. Five rats were raised in one cage after weaning, and they were separated when they were 60 days old in order to perform the behavior test. This study was performed with the approval of the local ethical committee, and all the experiments were performed according to the National Institutes of Health Guide for the Care and Use of Laboratory Animals.

### 2.2 Generation of experimental complex FSs

Day of birth was considered as postnatal day 0 (P0). Experimental FSs were induced as we previously described in rat pups on P10.^9^, ^13^, ^14^ Briefly, the body temperature of the pups was raised in a chamber with an ambient temperature of 42‐44°C. Core temperature was measured at baseline (34.1 ± 0.7°C) and seizure onset (40.3 ± 0.7°C). Pups were moved to a cool surface for 2 minutes once seizure was evoked and then returned to the chamber. The behavioral seizures induced by hyperthermia were correlated with EEG seizures and stereotyped, consisting of sudden movement arrest followed by facial automatisms (chewing), forelimb clonus, and tonic flexion of the body, often associated with a loss of postural control. The hyperthermia was maintained for ~90 minutes (typically 40‐45 minutes of seizures) for 10FS and ~60 minutes (typically 15‐18 minutes of seizures) for 4FS rats. After hyperthermia, pups were weighed and moved to a cool surface until core temperature fall within the normal range for age, and then returned to the home cage.

The rat pups were divided into four groups as follows: (a) four FSs within 30 minutes (4FSs); (b) ten FSs within 60 minutes (10FSs); (c) hyperthermia control (H), which subjected to ten episodes of hyperthermia along with 10FSs group, but seizures were prevented by pretreatment of pentobarbital (20 mg/kg, ip); and (d) controls (CON), which were removed from the cage along with FSs groups during the experiment but were maintained in the normal environment.

The body weight of FSs rats and control rats were measured every day from 7 days old to 60 days old. Then, the behavioral experiments were performed at about P60, and the rats were tested over few days. The same cohorts of rats were used for different behavioral tests to minimize the use of the number of rats. The interval time of two behavioral tests was 1‐2 weeks. We used rats from different mothers to perform the same behavioral tests (about 2‐3 L from the same mother).

### 2.3 Offspring generation

Sixty‐day‐old F0 generation rats were allowed to mate. F1 generation rats were born when the F0 rats of ~90 days old were used. Then F1 generation rats were mated to have their offspring (F2 generation). To reveal which parent contributed to the transgenerational transmission, FSs females were mated with non‐FSs males and vice versa.

### 2.4 Cross‐fostering

All litters were cross‐fostered on P2 according to age, dam availability, and birth date concordance.^15^ Litters were culled to 7‐8 pups for homogeneity. Four groups were obtained as follows: (a) pups born from FSs dams but were adopted by normal dams (FS (F1)‐CON (F0)); (b) pups born from FSs dams and were adopted by FSs dams (FS (F1)‐FS (F0)); (c) pups born from normal dams and were adopted by FSs dams (CON (F1)‐FS (F0)); (d) pups born from normal dams and were adopted by normal dams (CON (F1)‐CON (F0)).

### 2.5 Locomotor activity

Locomotor activity was recorded in an open‐field arena with a camera connected to a tracking system. The protocol was simplified from previous report^17^: P60 rats were placed individually in the center of an open circular arena (100 × 100 × 40 cm) located in a sound‐attenuated, temperature controlled room. The rats were maintained in the arena for 30 minutes. Their exploratory activities were videotaped, and behavior analyses were performed by an expert observer without knowledge of the treatments given.

### 2.6 Morris water maze

On around P60, rats underwent the Morris water maze to assess hippocampus‐dependent spatial memory.^16^ In brief, on days 1 to 4, rats were given 24 training sessions (six per day) to escape onto the submerged platform. On day 5, platform was removed, and rats were placed in the quadrant opposite to the previous platform position. The rats were allowed 60 seconds of free swimming. The time in target quadrant and the number of crossings in the target area were recorded.

### 2.7 Inhibitory avoidance task

The single‐trial inhibitory avoidance task, another hippocampus‐dependent behavior test, was used to measure different phases of memory in adult rats. In the training phase, rat was placed in the illuminated compartment. The door was opened 2 minutes later and was closed when the rat entered the dark compartment. Rat was given a 1.0 mA/s shock and then was removed from the alley and returned to its home cage. The rat was placed in the illuminated compartment 24 hours later, and the latency to step into the dark compartment was recorded as the measure of retention performance. Rat that did not enter the dark compartment within 600 seconds was removed from the alley.

### 2.8 Contextual fear conditioning

In a typical experiment, the rat was placed in a fear conditioning apparatus.^17^After the initial adaptation, a foot shock (1 seconds, 0.5 mA) was given, and this process repeats for three times. Rat was placed in the same apparatus 24 hours after training. Freezing times in response to representation of the context were measured every 5 seconds in 5 minutes.

### 2.9 Enrichment protocol

Siblings were divided equally between test and control cohorts. Enriched environment (EE) included an enriched cage (60 × 60 × 60 cm) containing plastic play tubes, cardboard boxes, running wheel, various pet toys, and nesting material that were all changed or rearranged every other day to provide novel stimulation. The EE group consisted of 21‐day‐old FSs or control rats that explored the enriched cage for 6 hours per day for 14 days. Age‐matched animals were housed three to four per cage in standard cages containing only pine chip bedding. The rat pups were randomly divided into four groups as follows: (a) control rats in normal environment (CON‐N); (b) control rats in enriched environment (CON‐E); (c) 10FSs rats in normal environment (10FSs‐N); (d) 10FSs rats in enriched environment (10FSs‐E).

### 2.10 DNMT inhibitor treatment

Zebularine (Sigma, St. Louis, MO, USA, Z4775) was dissolved in 10% DMSO and diluted to a concentration of 2 mg/mL in sterile saline. 5‐Aza‐2′‐deoxycytidine (Sigma, A3656) was dissolved in 0.8% acetate and diluted to a concentration of 1 mg/mL in sterile saline. Rats of P10 were given 0.1 mL DNMT inhibitor (ip) immediately after FSs and then daily for the following 5 days.^18^

### 2.11 Protein extraction

Protein extractions were performed from adult male or female rats (P60), which have not yet been tested in water maze test, inhibitory avoidance task or contextual fear conditioning. Rats were deeply anesthetized with ethyl ether and perfused transcardially with 0.9% saline. Animals were decapitated, and the hippocampus, testis or ovaries were quickly isolated. The collected tissues were homogenized in RIPA buffer (pH 7.5, in mmol/L; 20 Tris‐HCl, 150 NaCl, 1 EDTA, 1% Triton‐X100, 0.5% sodium deoxycholate, 1 PMSF, and 10 μg/mL leupeptin). Centrifuge for 30 minutes at 12000 rpm and collect the clear supernatant into a new tube. Determine protein concentration with a dilution.

### 2.12 Western blotting

Protein extracts were separated by SDS‐PAGE on a 7.5% resolving gel with a stacking gel and transferred onto nitrocellulose membrane.Blots were placed in 5% skim milk for 1 hours at room temperature, and then incubated with primary antibody (diluted in TBS/0.05% Tween) overnight at 4°C. Subsequently, the blots were washed and probed with the respective horseradish peroxidase‐conjugated secondary antibody (Odyssey, LI‐COR, MultiSciences, Hangzhou, China, 1:5000 dilution) for 2 hours at room temperature. The immunoreactive bands were visualized using the ECL detection reagent (Millipore, Billerica, MA, USA).^19^, ^20^ The following primary antibodies were used: anti‐DNMT1 (1:1000, Cell Signaling Technology, Danvers, MA, USA, D63A6), anti‐DNMT3A (1:1000, Abcam, Cambridge, UK, ab113430), anti‐DNMT3B (1:1000, Abcam, ab79822), anti‐Reelin (1:1000, Abcam, ab18570), anti‐protein phosphatase 1 (PP1) (1:1000, Cell Signaling Technology, #2582), anti‐β‐Actin (1:1000, Santa Cruz Biotechnology, Dallas, TX, USA, sc47778).

### 2.13 RT‐PCR

RNA extractions were performed from adultmale or female rats, which have not yet been tested in water maze test, inhibitory avoidance task or contextual fear conditioning. RNA was isolated from the hippocampus using Trizol (Invitrogen Carlsbad, CA, USA). First‐strand cDNA synthesis was carried out using 2 μg total RNA with reverse transcriptase. SYBR green was used to monitor amplification of template with primers on a real‐time thermal cycler.^21^ The following PCR primers were used for RT‐PCR analysis: DNMT1: forward 5′‐GGGTCTCGTT CAGAGCTG and reverse 5′‐GCAGGAATTCATGCAGTAAG; DNMT3A: forward 5′‐CAGCGTCACACAGAAGCATATCC and reverse 5′‐GGTCC TCACTTTGCTGA ACTTGG; DNMT3B: forward 5′‐GAATTTGAGCAGCCCAGGTTG and reverse 5′‐T GAAGAAGAGCCTTCCTGTGCC^22^; reelin: forward 5′‐AAAGGGAGATTGGGTG ACG and reverse ACGTGCTTCTGGATGGTTTC; PP1: forward TCCATGGAGCA GATTAGACG and reverse GCTTTGGCAGAATTGCGG; β‐Actin: forward 5′‐G TGGGCCGCTCTAGGCACCAA and reverse 5′‐CTCTTTGATGTCACGCACGATT TC.

### 2.14 DNA methylation assay

DNA extractions were performed from adult male or female rats, which have not yet been tested in water maze test, inhibitory avoidance task or contextual fear conditioning. Purified DNA was then processed for bisulfite modification (CpGenome DNA modification kit; Chemicon Billerica, MA, USA). Semiquantitative real‐time PCR was used to determine the DNA methylation status of the *reelin* and *PP1* genes. Methylation‐specific PCR primers were designed according to previous research.^23^

Detection of unmethylated *reelin* DNA was performed using the following primer: forward (5′‐TGTTAAATTTTTGTAGTATTGGGGATGT‐3′) and reverse (5′‐TCCTTAAAATAATCCAACAACACACC‐3′). Detection of methylated *reelin* DNA was performed using the following primer: forward (5′‐GGTGTTAAATTTTT GTAGTATTGGGGAC‐3′) and reverse (5′‐TCCTTAAAATAATCCAACAACACGC‐3′). Detection of unmethylated *PP1* DNA was performed using the following primer: forward (5′‐GAGGAGAGTTTGGTGTTTATAA GATGGT‐3′) and reverse (5′‐TCC TCCAAAAACTCAACTCAAACAA‐3′). Detection of methylated *PP1* DNA was performed using the following primer: forward (5′‐GGAGAGTTTGGTGTTTATAA GATGGC‐3′) and reverse (5′‐CGAAAACTCGACTCGAACGA‐3′). Samples were normalized to β‐tubulin 4 using following primer: forward (5′‐GGAGAGTAAT ATGAATGATTTGGTG‐3′) and reverse (5′‐CATCTCCAACTTTCCCTAACCTAC TTAA‐3′).

### 2.15 Statistical analysis

Data were expressed as mean ± SEM. Two‐tailed unpaired t test was used for two‐group comparison, and One‐way ANOVA (analysis of variance) with Dunnett's post hoc test was used for multiple comparisons. A two‐tailed *P* < 0.05 was considered statistically significant.

## 3 RESULTS

### 3.1 Transgenerational transmission of long‐term memory deficits

**Figure 1:**
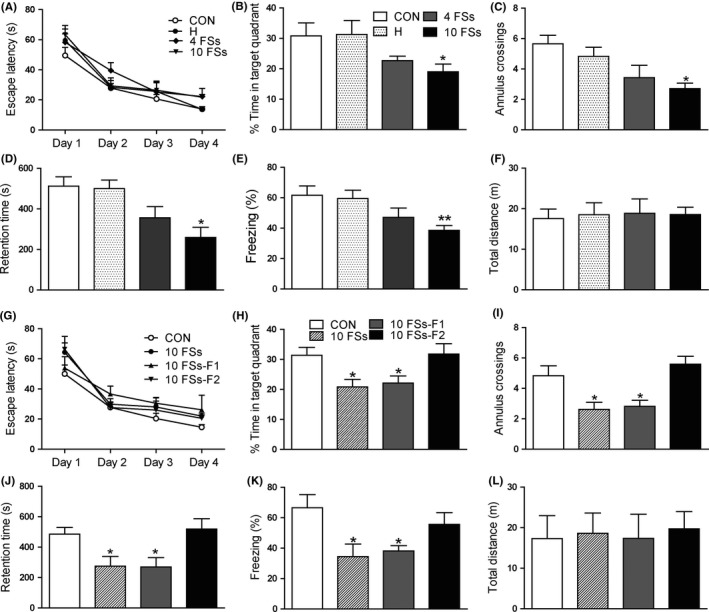
Memory deficits after prolonged FSs are transmitted transgenerationally. (A,B,C) Morris water maze task. A, During the four days of training, the escape latencies of four groups decreased gradually. B, 10FSs rats spent significantly less time in the target quadrant than did controls when the platform was removed. C, The numbers of annulus crossings were also less in 10FSs group than control rats. n = 8 for all group; *P < 0.05 compared with CON group. D, 10FSs group showed significantly shorter retention time than controls 24 hours after training in inhibitory avoidance task (n = 8 for all group, *P < 0.05 compared with CON group). E, In contextual fear conditioning, 10FSs rats showed significantly lower levels of freezing than control rats 24 hours after training (n = 8 for all group, **P < 0.01 compared with CON group). F, The locomotor activity of four groups had no significant difference (n = 8 for all group). G, During the four days of training, the escape latencies of four groups decreased gradually. H, Offspring of 10FSs rats (10FSs‐F1) spent significantly less time in the target quadrant than controls although they had never experienced FSs. I, The numbers of annulus crossings were also less in 10FSs rats and their offspring (F1) than control rats (n = 8 for all group, *P < 0.05 compared with CON group). J, 24 hours after training, 10FSs rats and their offspring showed significantly shorter retention time than controls (n = 8 for all group, *P < 0.05 compared with CON group). K, 10FSs rats and their offspring showed significantly lower levels of freezing in contextual fear conditioning (n = 8 for all group, *P < 0.05 compared with CON group). L, The locomotor activity of four groups had no significant difference (n = 8 for all group). One‐way ANOVA followed by Dunnett's multiple comparisons test was used, Error bars indicated SEM

As shown in Figure 1, a gradual decrease in escape latency in the Morris water maze task was observed in all rats (Figure 1A). In the probe test, the 10FSs group spent less time in the target quadrant than control group, but there were no significant differences among CON, H, and 4FSs groups (Figure 1B). The number of annulus crossings, an index of memory precision, showed similar tendency (Figure 1C). In the inhibitory avoidance task, the 10FSs group showed shorter retention latency and lower levels of freezing than control rats in contextual fear conditioning (Figure 1D,E). In order to exclude the influence of different locomotivity among four groups, the open‐field test was done. Here, we found that there were no significant differences in the distances moved among four groups (Figure 1F).

Interestingly, the unaffected offspring of 10FSs rats spent less time in the target quadrant and showed less number of annulus crossings in Morris water maze test (Figure 1H,I), although the escape latencies of four groups decreased gradually during the four days of training without significant difference (Figure 1G). Likewise, the 10FS‐F1 rats showed shorter retention time in inhibitory avoidance task (Figure 1J) and much lower levels of freezing in contextual fear conditioning (Figure 1K) than control rats, indicating the memory deficit induced by FSs was transmitted to their unaffected F1 generation. However, the harmful effect of FSs on memory was lost in the F2 generation, as their performances in Morris water maze, inhibitory avoidance task, and contextual fear conditioning were not statistically different from that of control rats. In open‐field test, there were no significant differences in the distances moved among four groups (Figure 1L).

In addition, to check whether FS animals have developmental problems, we have measured the body weight of FS rats and normal rats from 7 days old to 60 days old. We also found no significant difference of body weight between FS rats and normal rats (Figure S1).

### 3.2 Transgenerational transmission of long‐term memory deficits is through the mother

**Figure 2:**
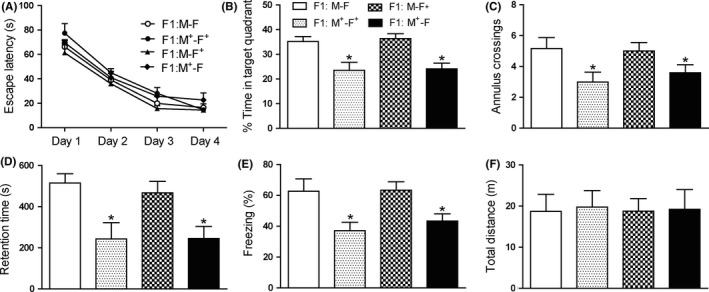
Transgenerational transmission of memory deficits is through the mother. (A,B,C) Morris water maze task. A, During the four days of training, the escape latencies of four groups decreased gradually. B, Offspring of FSs mothers and FSs fathers (F1:M^+^‐F^+^) or FSs mothers and non‐FSs fathers (F1:M^+^‐F) had similar performance in test, but they spent significantly less time in the target quadrant than controls (F1:M‐F), while offspring of FSs fathers and non‐FSs mothers (F1:M‐F^+^) had similar performance with controls. C, The number of annulus crossings was also less in offspring of FSs mothers than controls (n = 8 for all group, *P < 0.05). D, 24 hours after training, offspring of FSs mothers showed significantly shorter retention time than controls (n = 8 for all group, *P < 0.05). E, In contextual fear conditioning, offspring of FSs mothers showed significantly lower levels of freezing than control pups (n = 8 for all group, *P < 0.05). F, The total distance of four groups had no significant difference (n = 8 for all group). One‐way ANOVA followed by Dunnett's multiple comparisons test was used. Error bars indicated SEM. *Significant vs CON. M: mother; F: father; ^+^treated with 10FSs

Interestingly, we found that only offspring whose mother experienced FSs displayed long‐term memory deficits. Offspring of FSs mother and non‐FSs father displayed memory deficits in Morris water maze (Figure 2A‐C), inhibitory avoidance task (Figure 2D), and contextual fear conditioning (Figure 2E), which were equal to offspring of FSs mother and FSs father. In addition, offspring of FSs father and non‐FSs mother displayed similarly memory ability as control rats (Figure 2).

In order to exclude the influence of maternal behavior on the memory performance of their offspring,^24^ we next used cross‐fostering. As shown in Figure S2, control pups adopted by control mothers or FSs mothers displayed similar ability of memory in Morris water maze, inhibitory avoidance task, and contextual fear conditioning (Figure S2B‐E). While pups born from FSs rats adopted by control mothers or FS mothers showed memory deficits in three memory tasks (Figure S2B‐E), indicating maternal behavior is not the cause of memory deficits.

### 3.3 DNA methyltransferase activity is necessary for memory deficits

**Figure 3:**
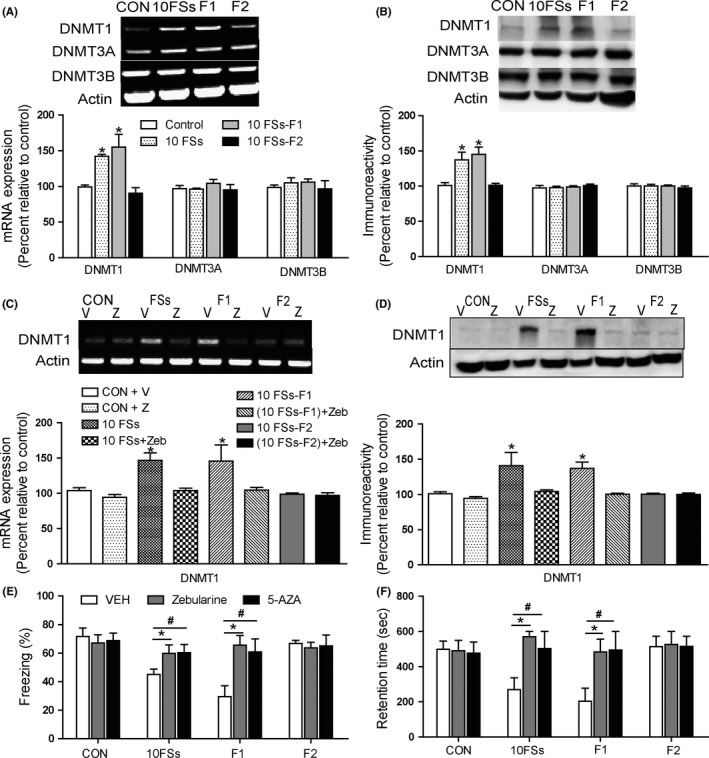
DNMT mRNA activity is reasonable for the transgenerationally memory deficits after prolonged FSs. (A‐B) DNMT1 mRNA (A) and protein (B) in the hippocampus were upregulated in 10FSs rats and their offspring (F1) but not F2 generation (F2) comparing to control rats (n = 3 for all groups, *P < 0.05). C and D, When given immediately after 10FSs and then daily for the following 5 days, zebularine decreased the mRNA (C) and protein (D) level of DNMT1 in adult 10FSs rats and their offspring (n = 3 for all groups, *P < 0.05). E, DNMT inhibitor increased the freezing behavior in 10FSs rats and their offspring (n = 8 for all groups, *P < 0.05，^#^ *P* < 0.05). F, DNMT inhibitor increased the retention time in 10FSs rats and their offspring (n = 8 for all groups, *P < 0.05，^#^ *P* < 0.05). V, VEH: vehicle；Z, zebularine. One‐way ANOVA followed by Dunnett's multiple comparisons test was used. Error bars indicated SEM

We next test whether DNA methylation contributes to the transgenerational transmission of memory defect induced by FSs. We assayed levels of three DNMT subtypes, DNMT 1, 3A, and 3B in the hippocampus of adult rats. The 10FSs rats but not hyperthermia (H) or 4FSs rats displayed an increase in DNMT1 mRNA (Figure S3A) and DNMT1 protein (Figure S3B) relative to control rats. Interestingly, offspring of 10FSs rats also displayed an increase in DNMT1 mRNA and protein expression (Figure 3A,B), but not DNMT3A or DNMT3B, in the hippocampus compared with control rats. However, the DNMT1 mRNA or protein expression of F2 generation did not show obvious changes (Figure 3A,B). Furthermore, the level of DNMT1 increased significantly in offspring of FSs mothers and non‐FSs fathers but not offspring of FSs fathers and non‐FSs mothers (Figure S4A,B). We next administered two distinct DNMT inhibitors, 5‐Aza‐2′‐deoxycytidine (5‐AZA) or zebularine, to rat pups immediately after FSs and then daily for the following 5 days. Zebularine decreased levels of DNMT1 mRNA and protein in 10FSs rats and their F1 offspring (Figure 3C,D). Meanwhile, performances of freezing and retention time in 10FSs rats and their offspring were improved by zebularine or 5‐AZA (Figure 3E,F).

### 3.4 Hypermethylation of *reelin* gene is involved in memory deficits after FSs

To further confirm the role of DNMTs in memory defect, we focus on the genes that were related with memory and could be modified by DNA methylation. We have explored some genes that are closely related with memory function, such as KCC2, CREB, BDNF, PP1, and reelin.^25^, ^26^ However, for BDNF, PP1, or CREB, we found no change after FSs. For KCC2, although its level was lower in FSs rats and their offspring than control rats, its methylation state had no change (data not shown). Therefore, based our preliminary data, we chose reelin, which had a reduction after FSs and easily be adjusted by DNA methylation in our experiment.

**Figure 4:**
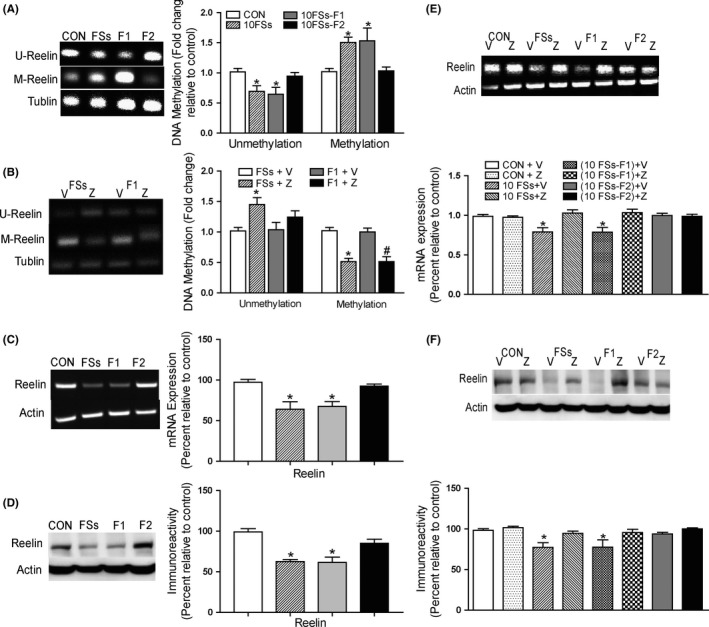
DNA methylation leads to transcriptionally inhibition of a memory promoting gene *reelin*. A, Increased DNA methylation of reelin gene in the hippocampus of 10FSs rats and their offspring (10FSs‐F1) compared to controls (n = 3 for all groups, *P < 0.05); U, unmethylated; M, methylated; Tub, β‐tubulin 4. B, Levels of methylated reelin gene decreased after zebularine (Z) treatment in FSs rats and their offspring (F1 rats), n = 3 for all groups, *P < 0.05. C and D, Levels of reelin mRNA (C) and protein (D) decreased in the hippocampus of 10FSs rats and their offspring (n = 3 for all groups, *P < 0.05). E and F, Zebularine treatment increased mRNA (E) and protein (F) levels of reelin (n = 3 for all groups, *P < 0.05). One‐way ANOVA followed by Dunnett's multiple comparisons test was used. Error bars indicated SEM. *Significant vs control

Next, we employed methylation‐specific real‐time PCR to examine the level of *reelin* gene. As shown in Figure 4A, 10FSs adult rats (FSs), and their offspring (F1) showed a significant increase in methylated *reelin* gene (M‐Reelin) and concomitant reduction in unmethylated *reelin* gene (U‐Reelin) relative to controls (CON). We also found that zebularine attenuated *reelin* methylation in 10FSs rats and their offspring relative to vehicle controls (Figure 4B). For 10FSs rats and their offspring, the hypermethylation of *reelin* gene finally led to lower levels of transcription and expression in the hippocampus comparing to control rats (Figure 4C,D). We also found the hypermethylated *reelin* gene (Figure S5A) with decreased expression of reelin mRNA (Figure S5B) and protein (Figure S5C) in FSs mothers and non‐FSs fathers but not offspring of FSs fathers and non‐FSs mothers. In addition, zebularine increased the transcription and expression of *reelin* gene in 10FSs rats and their offspring but not control and F2 generation (Figure 4E,F), whereas the *PP1* gene, which inhibits synaptic plasticity and memory,^28^ showed no significant differences of unmethylation or methylation levels among control rats, 10FSs rats, F1 rats, and F2 rats (Figure S6A). The methylation state of PP1 remained unaltered when FSs were coupled with zebularine treatment (Figure S6B). The levels of PP1 mRNA and protein in the hippocampus of 10FSs adult rats and their offspring were equal to that of control rats (Figure S6C,D).

### 3.5 Juvenile‐enriched environment rescues the memory deficit in FSs rats

**Figure 5:**
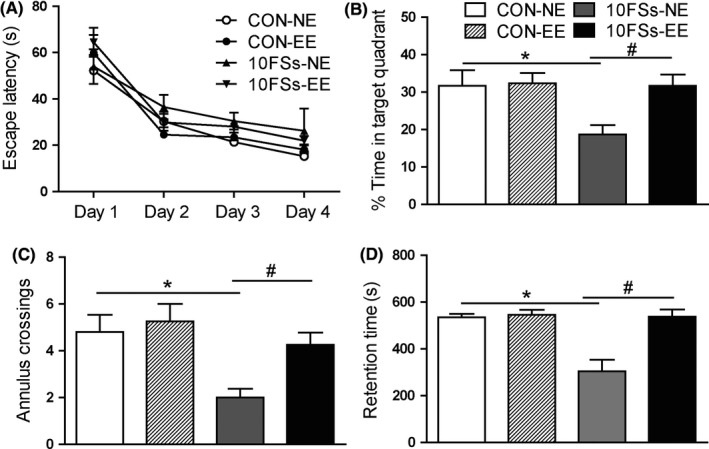
Early EE rescues memory deficits in adult FSs rats. A, In morris water maze, during the first 4 days of training, a gradual decrease in escape latency was observed for all rats (n = 8 for all groups). B, On the probe test, the 10FSs rats receiving early enrichment (10FSs‐EE) spent significantly more time in the target quadrant than those reared in normal environment (10FSs‐NE), but there was no significant difference between control rats reared in normal environment (CON‐NE) and that receiving early enrichment (CON‐EE) (n = 8 for all group, *P < 0.05, ^#^ *P* < 0.05). C, The numbers of annulus crossings were more in FSs‐EE group than FSs‐NE group (n = 8 for all group, *P < 0.05, ^#^ *P* < 0.05). D, In inhibitory avoidance task, the 10FSs‐EE group showed significantly longer retention time than FSs‐NE group (n = 8 for all group, *P < 0.05, ^#^ *P* < 0.05). One‐way ANOVA followed by Dunnett's multiple comparisons test was used. Error bars indicated SEM. *Significant vs CON‐NE. ^#^Significant vs 10FSs‐NE

As DNMT inhibitors were usually used for cancer treatment but not for FSs, which might have unpredictable side effects on infants,^29^ we aimed to search an effective and harmless treatment that could also rescue the future memory deficits induced by prolonged FSs. Here, we investigated the effect of an enriched environment (EE) protocol during weaning period on adult memory performance. In the Morris water maze, a gradual decrease in escape latency was observed for all rats during the first 4 days of training (Figure 5A). In the probe test, the EE‐experienced 10FSs rats spent significantly more time in the target quadrant than normal environment (NE)‐experienced 10FSs rats, but there was no significant difference between EE‐experienced 10FSs rats, EE‐experienced and NE‐experienced control rats (Figure 5B). Similarly, the number of annulus crossings of EE‐experienced 10FSs rats was more than NE‐experienced littermates (Figure 5C). In inhibitory avoidance task, the EE‐experienced 10FSs rats showed significantly longer retention time than NE‐experienced littermates (Figure 5D). These results indicated that early enrich environment treatment could rescue the adult memory deficits induced by infantile FSs.

## 4 DISCUSSION

One of the most important findings of our study was that memory deficits after infantile FSs could be transmitted to next generation. Ten FSs rats and their unaffected offspring performed significantly worse in Morris water maze, inhibitory avoidance task, and contextual fear conditioning, which were in accordance with the alteration of DNA methylation in the hippocampus. These memory deficits and DNA methylation only occurred after severe FSs but not 4FSs, suggesting that the danger of FSs was intensity‐dependent. Interestingly, these memory deficits and DNA methylation induced by 10FSs can be transgenerational transmitted to next generation. Another significant characteristic was that the memory deficits were passed on to subsequent generation through the mother. Therefore, our study demonstrated the significant effect of severe FSs on later adult memory and uncovered the crucial characteristics.

Previous studies found that childhood maltreatment changed neural structure and function, further rendering them more susceptible to later cognitive deficits.^30^, ^31^ In these studies, the cognitive deficit transgenerationally transmitted from mothers to their unaffected offspring through after‐birth event, such as licking/grooming. However, in our study, we found that memory deficits were transmitted to offspring even if they were raised by non‐FSs foster mothers. Our data at least indicated that the crucial event for the change of cognitive network may occur before the birth of unaffected offspring. In addition, though we could not absolutely exclude higher perinatal stress in the 10FS mother compared to the 10 hyperthermia exposure mother, we do found that the mental status of 10FSs mothers was equal to that of hyperthermia only mothers by observing the behavior of diet and rearing during perinatal stage. In this case, we supposed that the memory deficits of offspring were not mainly caused by the phenotype of their perinatal mothers; change in the germ stage was one of the possibilities. As previously reported, exposure to environmental stress induced epigenetic alterations in germ cells, which might then affect the phenotype of offspring to major depressive disorder.^32^ In this case, it was deducible that exposure to severe FSs induced by environmental hyperthermia might produce epigenetic alterations in germ cells of affected mothers, which might then affect the phenotype of offspring to memory deficits.

DNA methylation, a strong epigenetic marker for the transgenerational inheritance, is altered by environmental factors at specific genes and maintains across generation,^33^, ^34^ which is associated with multiple brain diseases and psychiatric disorders.^35^, ^36^ Our findings implicated a possible mechanism of the transmission of acquired diseases by showing that FSs altered DNA methylation patterns. Although DNA methylation always existed in the process of development, we found that DNA methylation levels were upregulated right after 10 episodes of FSs in pups, and this increase of methylation levels was then maintained to adult (data not shown). In our study, it is likely that the increase of DNA methylation was mainly induced by 10FSs but not elevated with age. In addition, these altered DNA methylation patterns are faithfully maintained by DNMT1 during DNA replication to ensure epigenetic inheritance across generation.^37^ In our study, the DNA methylation patterns were enhanced and regarded as DNA hypermethylation. We found that DNA hypermethylation after FSs was detrimental to cognitive development after FSs, which was inconsistent with the previous study that DNA methylation displayed an essential effect on neural development. Our result was supported by the report that hypermethylation of the *reelin* gene provides a molecular basis for the *reelin* gene hypoactivity in schizophrenia.^38^ Interestingly, DNA hypomethylation restricted to the murine forebrain induces cortical degeneration and impairs postnatal neuronal maturation,^39^ which suggests that DNA hypomethylation can also be harmful. Therefore, it is likely that the level of DNA methylation needs a balance, too much or too less is both harmful. Our study at least indicated that, although DNA methylation was required in normal development of brain, excessively increased DNA methylation after FSs conversely inhibits the cognitive development.

Through upregulation of DNA methylation levels, the hippocampus adopts a way for the specific regulation of genes. The certain genes must be regulated by hypomethylation or hypermethylation to induce memory impairment. In regard to DNA hypermethylation, we focused on reelin, a gene that promotes synaptic plasticity and memory.^40^ In regard to DNA hypomethylation, we focused on PP1. Here, we found that the hypermethylation of reelin contributed to inheritable memory deficits after serious FSs. It was reasonable as in our study, DNA hypermethylation was more than DNA hypomethylation, which attributed to the increase of DNMT1. The infantile FSs led to the DNA hypermethylation of many genes including reelin, which plays an important role in memory defects induced by FSs in our study. However, other genes could not be excluded. Furthermore, we were interested to find that the enriched environment rescued spatial memory in Morris water maze and long‐term memory in passive avoidance, suggesting that enriched environment was an effective modulation to rescue memory deficits. Recently, it has been reported that an enriched environment can attenuate surgery‐induced impairment of learning, memory, and neurogenesis.^41^ Because enriched environment training is easy to implement in clinic, it is proposed that if we apply enriched environment training on juvenile FSs patients, it may prevent the possible following memory deficient in adult and even their offspring. Thus, our study provided a perspective for searching the potential target of diagnoses and treatment of memory deficits induced by FSs in clinic.

In conclusion, these observations demonstrated that postnatal FSs persistently affect behavior and DNA methylation even in their offspring that never experience FSs. It significantly extends previous knowledge that DNA methylation in the brain is influenced by maternal care and illustrates the detrimental impact of early FSs. Our study also provided strategies to identify molecular targets for pharmacologic treatment of FSs‐induced cognitive disorders with epigenetic pathomechanisms.

## CONFLICT OF INTEREST

The authors declare no conflict of interest, and all the authors listed have approved the manuscript.
