# Supplementary material for: Prolonged febrile seizures induce inheritable memory deficits in rats through DNA methylation
Source: CNS Neurosci Ther. 2019 Jan 21;25(5):601–11. doi: 10.1111/cns.13088 (PMC6488897; doi:10.1111/cns.13088)
Supplement: Supplementary file 1 [file CNS-25-601-s001.docx]

**Supplementary information**

**

**

**Supplementary Figure 1: Body weight of FS rats and normal rats.** The body weight of FS rats and normal rats from 7 days to 60 days old (n=8 for all group). Error bars indicated SEM.





**Supplementary Figure 2:** **Cross-fostering.** (**a,b,c**) Morris water maze task. (**a**) During the four days of training, the escape latencies of four groups decreased gradually (n=8 for all group). (**b**) F1 pups of FSs rats that were adopted by either control mothers (FS (F1)-CON (F0)) or FSs mothers (FS (F1)-FS (F0)) had similar performance in test, but they spent significantly less time in the target quadrant than controls pups (CON (F1)-CON (F0) and CON (F1)-FS (F0)) (n=8 for all group, **P*<0.05, **^#^***P*<0.05). (**c**) The number of annulus crossings was also less in FS (F1) pups than control pups no matter they were raised by FSs mother or control mother (n=8 for all group, **P*<0.05, **^#^***P*<0.05). (**d**) 24 h after training, FS (F1) pups in FS dams or control dams showed significantly shorter retention time than control pups (n=8 for all group, **P*<0.05, **^#^***P*<0.05). (**e**) In contextual fear conditioning, FS (F1) pups showed significantly lower levels of freezing than control pups 24 h after training (n=8 for all group, **P*<0.05, **^#^***P*<0.05). (**f**) The total distance of four groups had no significant difference. Error bars indicated SEM. One-way ANOVA followed by Dunnett's multiple comparisons test was used. *****Significant versus CON (F1)-CON (F0). **^#^**Significant versus CON (F1)-FS (F0). Error bars indicated SEM.


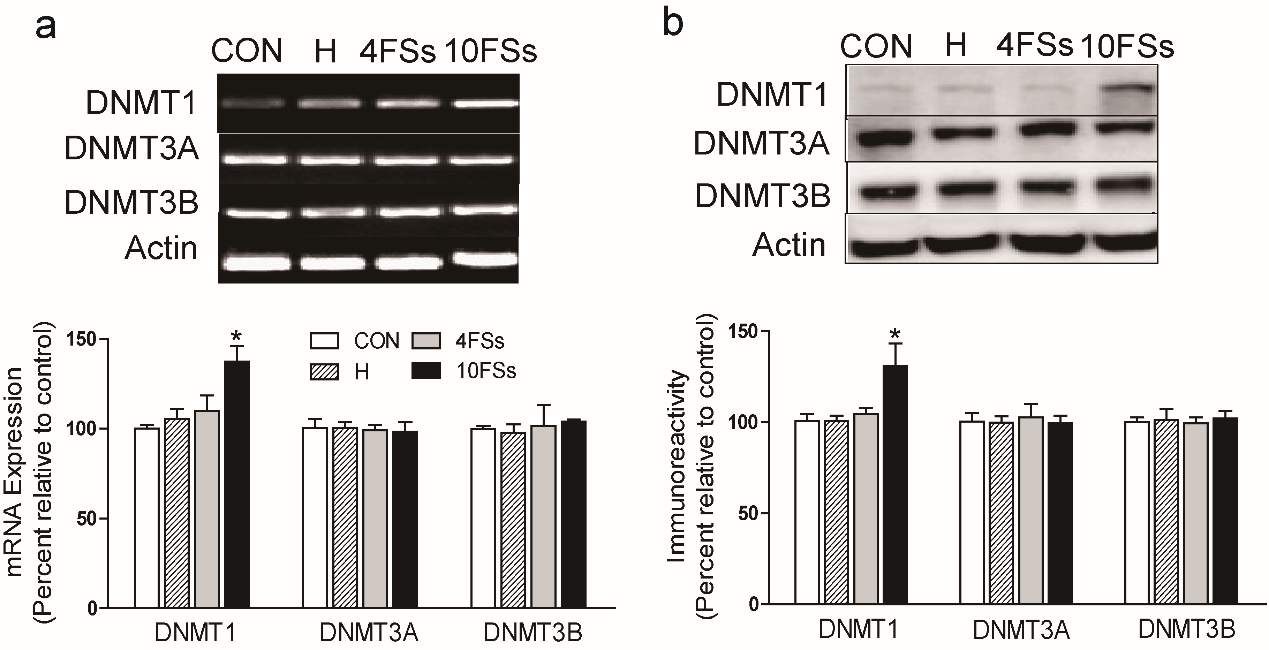


**Supplementary Figure 3:** **DNA methylation participates in the seizure intensity-dependent memory deficits after FSs. (a)** The DNMT1 mRNA was increased in hippocampus of 10 FSs rats but not 4 FSs and hyperthermia only rats comparing to control rats (n=3 for all groups, *P<0.05). **(b)** The DNMT1 protein expression was increased in hippocampus of 10 FSs rats but not 4 FSs and hyperthermia only rats comparing to control rats (n=3 for all groups, *P<0.05). One-way ANOVA followed by Dunnett's multiple comparisons test was used. Error bars indicated SEM.


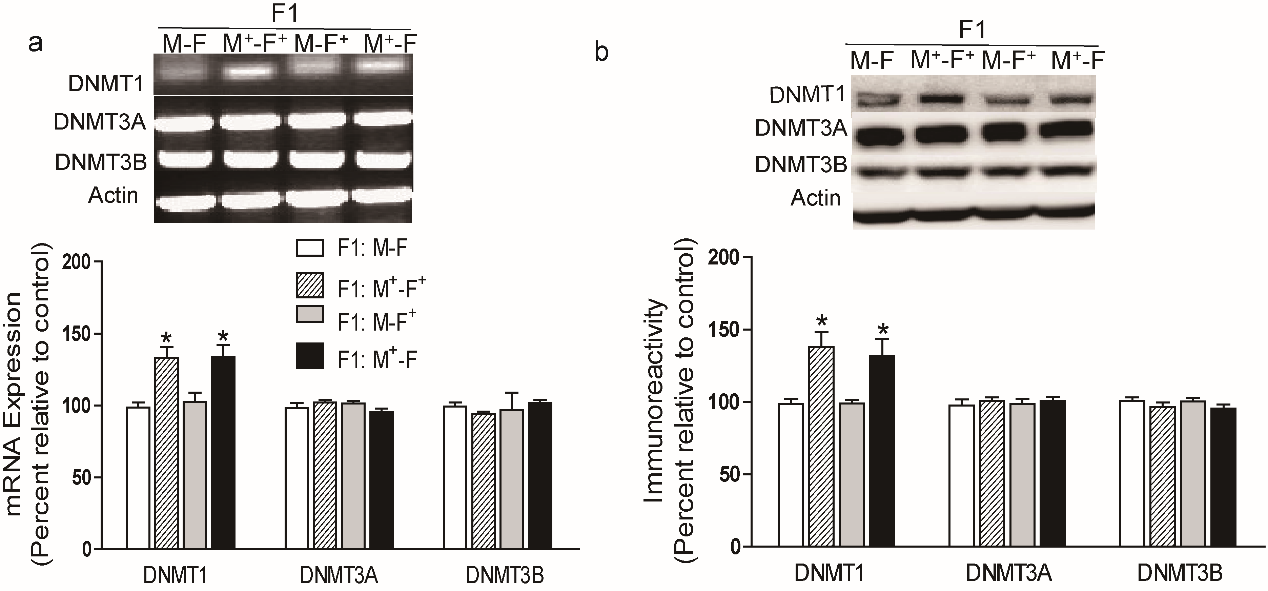


**Supplementary Figure 4: DNA methylation participates in the transgenerational transmission of memory deficits through the mother.** (**a-b**) DNMT1 mRNA **(a)** and protein **(b)** in the hippocampus were up-regulated only in offspring of FSs mothers (F1: M^+^-F^+^, F1: M^+^-F) comparing to controls (F1: M-F, n=3 for all groups). *Significant versus control. M: mother; F: father; + treated with 10 FSs. One-way ANOVA followed by Dunnett's multiple comparisons test was used. Error bars indicated SEM.


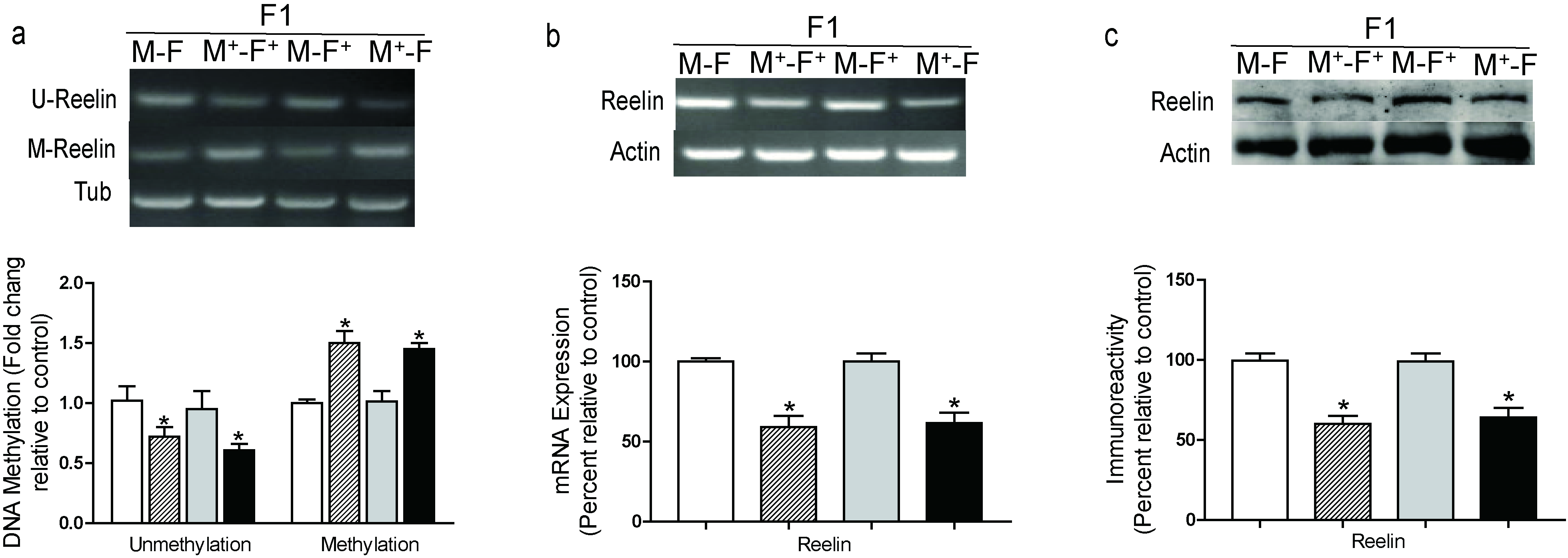


**Supplementary Figure 5: *Reelin* hypermethylation participates in the transgenerational transmission of memory deficits through the mother.** **(a)** Increased DNA methylation of *reelin* in the hippocampus of offspring of FSs mothers compared to controls and offspring of FSs fathers (F1: M-F+) (n=3 for all groups, *P<0.05); U, unmethylated; M, methylated; Tub, β-tubulin 4. **(b-c)** Levels of *reelin* mRNA **(b)** and protein **(c)** decreased in the hippocampus of offspring of FSs mothers (n=3 for all groups, *P<0.05). *Significant versus control. M: mother; F: father; + treated with 10 FSs. One-way ANOVA followed by Dunnett's multiple comparisons test was used. Error bars indicated SEM.


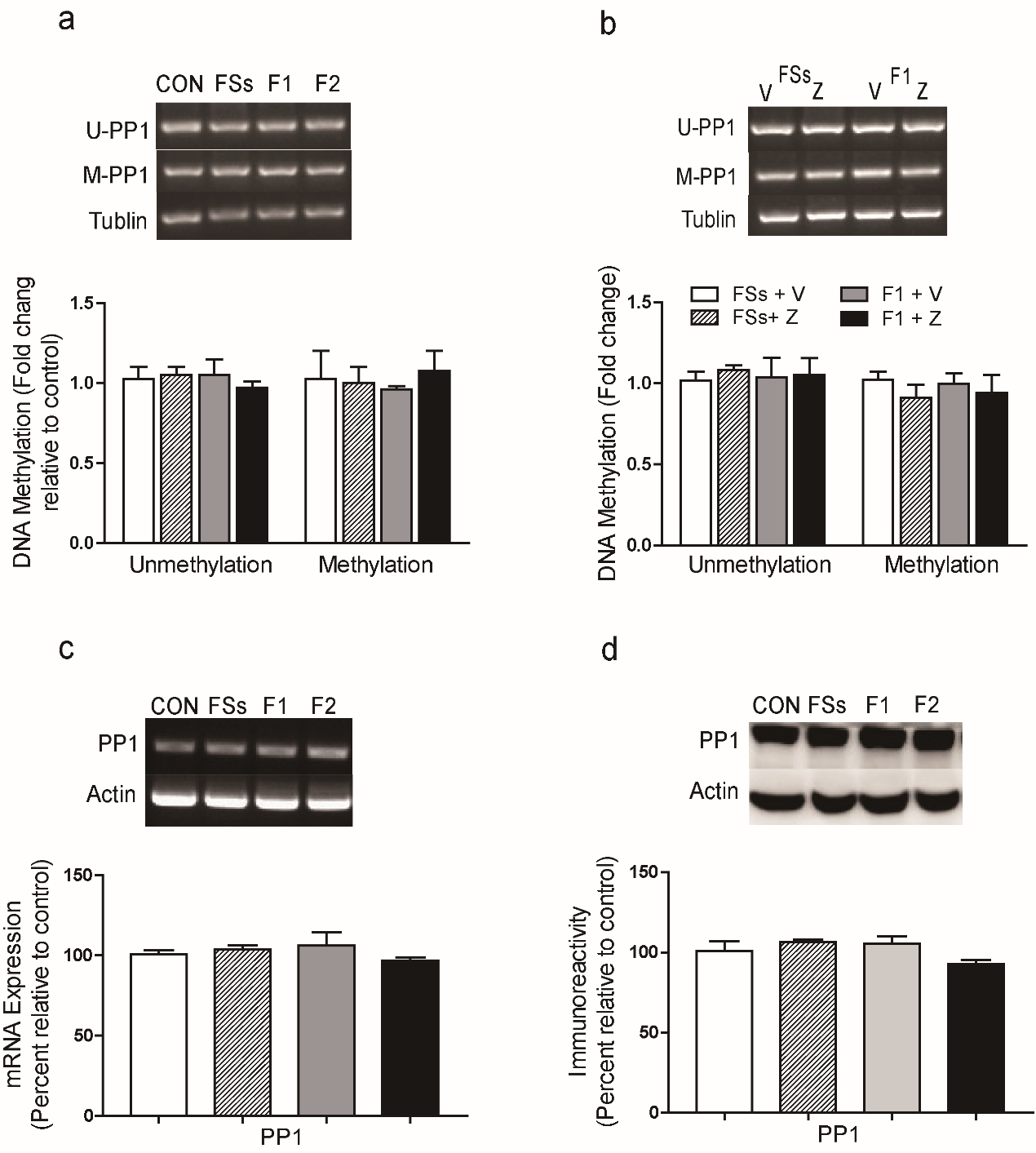


**Supplementary Figure 6: DNA methylation does not influence transcriptionally state of a memory inhibitor gene *PP1*.** **(a)** The DNA methylation of *PP1* had no difference among control rats (CON), 10FSs rats (FSs), F1 rats and F2 rats (n=3 for all groups). **(b)** The methylation states of *PP1* gene in FSs rats and their offspring were not influenced by zebularine (n=3 for all groups, one way ANOVA followed by Dunnett's multiple comparisons test was used). **(c-d)** Levels of PP1 mRNA and protein were not changed in FSs rats and their offspring (n=3 for all groups). Error bars indicated SEM.
